# Hospital utilization rates following antipsychotic dose reduction in mood disorders: implications for treatment of tardive dyskinesia

**DOI:** 10.1186/s12888-020-02748-0

**Published:** 2020-07-11

**Authors:** Stanley N. Caroff, Fan Mu, Rajeev Ayyagari, Traci Schilling, Victor Abler, Benjamin Carroll

**Affiliations:** 1 Department of Psychiatry, Corporal Michael J. Crescenz VA Medical Center and the Perelman School of Medicine at the University of Pennsylvania, 3900 Woodland Avenue, Philadelphia, PA 19104 USA; 2 Analysis Group, 111 Huntington Ave, Boston, MA 02199 USA; 3 Teva Pharmaceuticals, 145 Brandywine Pkwy, West Chester, PA 19380 USA

**Keywords:** Tardive dyskinesia, Antipsychotic medication, Bipolar disorder, Major depressive disorder, Relapse, Healthcare burden

## Abstract

**Background:**

The relative benefits and risks of long-term maintenance treatment with antipsychotics have not been well studied in patients with bipolar disorder and major depressive disorder. For example, while antipsychotic dose reduction has been recommended in the management of serious side effects associated with antipsychotics, there is limited evidence on the impact of lowering doses on the course of underlying mood disorders.

**Methods:**

This retrospective cohort study analyzed the impact of antipsychotic dose reduction in patients with bipolar disorder or major depressive disorder. Medical claims from six US states over a 6-year period were analyzed for patients with ≥10% or ≥ 30% reductions in antipsychotic dose (cases) and compared using survival analyses with matched controls receiving a stable dosage. Outcomes included hospitalizations for disease-specific mood disorders, other psychiatric disorders and all-cause emergency room visits, and claims for tardive dyskinesia.

**Results:**

A total of 23,992 patients with bipolar disorder and 17,766 with major depressive disorder had a ≥ 10% dose reduction, while 19,308 and 14,728, respectively, had a ≥ 30% dose reduction. In multivariate analyses, cases with a ≥ 10% dose reduction had a significantly increased risk of disease-specific admission (bipolar disorder: hazard ratio [95% confidence interval], 1.22 [1.15–1.31]; major depressive disorder: 1.22 [1.11–1.34]), other psychiatric admission (bipolar disorder: 1.19 [1.13–1.24]; major depressive disorder: 1.17 [1.11–1.23]), all-cause admission (bipolar disorder: 1.17 [1.12–1.23]; major depressive disorder: 1.11 [1.05–1.16]), and all-cause emergency room visits (bipolar disorder: 1.09 [1.05–1.13]; major depressive disorder: 1.07 [1.02–1.11]) (all *P* <  0.01). Similar results were observed following an ≥30% dose reduction. Dose reduction was not associated with decreased claims for tardive dyskinesia.

**Conclusions:**

Patients with mood disorders who had antipsychotic dose reductions showed small but statistically significant increases in all-cause and mental health-related hospitalizations, which may lead to increased healthcare costs. These results highlight the need for additional long-term studies of the necessity and safety of maintenance antipsychotic treatment in mood disorders.

## Background

Bipolar disorder (BD) and major depressive disorder (MDD) have an estimated annual prevalence of 2.8 and 6.7%, respectively [1, 2]. Significant advances have been achieved in treating these disorders with a broad range of mood stabilizers, antidepressants, and electroconvulsive treatments. Several antipsychotics have also recently received approval for treatment of BD and MDD [3–12]. Although MDD is more prevalent in the population, antipsychotics are particularly effective and commonly started during the acute phase of mania in BD [13]. Despite the fact that evidence on the long-term effectiveness and safety of antipsychotics in these episodic and remitting disorders is limited, many patients remain on antipsychotics chronically and perhaps unnecessarily [3, 11, 12, 14–17].

Prolonged use of antipsychotics is associated with serious side effects, including tardive dyskinesia (TD) as one example [4–10]. Historically, patients with mood disorders were considered at high risk for TD when treated with first-generation antipsychotics (FGAs), although this is confounded by age, gender, and intermittent treatment, all of which are known risk factors for TD [10, 11]. By comparison, there are limited data on TD risk in patients with mood disorders receiving long-term treatment with second-generation antipsychotics (SGAs); although it appears to be diminished, the risk of TD remains clinically significant [11, 12, 18].

When side effects emerge during maintenance treatment with antipsychotics, management options include drug maintenance, discontinuation, switching, or dose reduction [5, 6, 12, 19–23]. For example, in patients with mood disorders in remission, maintenance treatment with antipsychotics may be unnecessary, and discontinuation may be reasonable to facilitate remission of TD or other side effects. In patients who require continued antipsychotic maintenance treatment, dose reduction has also been considered as an option once TD occurs. In a previous retrospective cohort study, we demonstrated that antipsychotic dose reductions resulted in significant increases in both all-cause and mental health-related hospitalizations, worsening the overall healthcare burden in schizophrenia patients [23]. However, there are no data examining the effect of dose reduction on symptoms of TD in mood disorders [20, 24, 25]. Even more important for clinical decision making, there is no evidence on the impact of antipsychotic dose reduction on the course of illness in patients with BD or MDD [14, 26].

To further address the gap in knowledge on the risks of dose reduction as a recommended treatment intervention for side effects of antipsychotic treatment, including TD, we used the same methodology from the previous study in schizophrenia [23] to conduct a retrospective matched cohort study to analyze the utilization of hospital-based resources resulting from ≥10% and ≥ 30% reductions in antipsychotic doses for patients with BD or MDD.

## Methods

### Study objective and data sources

This retrospective matched cohort study compared the risk of all-cause and mental health-related inpatient admissions and emergency room (ER) visits for patients with mood disorders who received a stable dose versus those who had a dose reduction of oral antipsychotic monotherapy. The methods used, which are briefly summarized here, were described in detail in a previous study of schizophrenia [23]. Identical selection and outcome criteria, procedures, and statistical analyses were employed, except in this study, the target population was patients with mood disorders.

This study used de-identified claims data from the most recent 6 years for 26.6 million Medicaid-eligible beneficiaries from Iowa, Kansas, Missouri, New Jersey, Mississippi, and Wisconsin from 2008 to 2017. The database complies with the privacy rules of the Health Insurance Portability and Accountability Act. This study was reviewed by the New England Independent Review Board on August 31, 2017 and granted an exemption from consent requirements.

### Patient selection

Eligible patients met the following inclusion criteria: ≥18 years of age on the index date; ≥1 diagnosis of BD or MDD in the most recent 6 years of data for each state; ≥2 fills of an oral antipsychotic prescription after the first BD or MDD diagnosis; ≥1 antipsychotic monotherapy treatment period that was ≥90 days with a stable oral dosage; and a baseline period of ≥6 months of continuous enrollment prior to the index date. Diagnoses were determined by ICD-9 and ICD-10 (*International Classification of Diseases*) codes recorded in the database.

Patients were excluded if they lived in New Jersey and turned 65 years of age after 2012 because they were eligibility for both Medicare and Medicaid, or if they received more than one oral antipsychotic concurrently.

Patients within the BD and MDD groups were identified as cases or controls based on whether they had a ≥ 10% antipsychotic dose reduction. Cases were defined as patients who had a stable dosage of oral antipsychotic monotherapy for ≥90 days and then experienced a ≥ 10% dose reduction from the stable dose. Controls, matched on age, sex, type or health plan, state, index drug (FGA or SGA), and index year, included patients who were on a stable dosage of monotherapy with a duration of ≥91 days and did not experience a ≥ 10% dose reduction. A subset of cases from the BD and MDD groups (and their matched controls) who had a dose reduction of ≥30% were selected for subgroup analyses. The ≥10% reduction was chosen to capture clinically significant but minor dose reductions, and ≥ 30% reduction was chosen as a moderate dose change for comparison. The full distribution of dose reductions by percentiles for selected drugs is listed in Additional file 4.

The index date was defined as the date of the initial dose reduction for cases and the first prescription fill after the first 90 days of a stable dose period for controls. The index drug was the oral antipsychotic being used on the index date. Patients were followed until 2 years after the index date, dose escalation, treatment switch/addition, an outcome event, or end of eligibility, whichever came first (study period).

### Study measures and outcomes

Patients were assessed during the baseline period or on the index date for demographics, index year, index drug, psychiatric comorbidity profile, Charlson Comorbidity Index (CCI), psychotherapy, psychiatric medication, and observed disease duration, defined as the first observed BD or MDD diagnosis date prior to the index date. The mean durations of follow-up were also reported.

The primary outcome measure of the study was all-cause inpatient admission and ER visits as well as admissions for disease-specific BD, MDD, or for other psychiatric diagnoses (for ICD-9 and ICD-10 codes see Additional file 1). For each analysis in which admissions associated with other listed psychiatric conditions were evaluated, admissions associated with disease-specific BD or MDD were excluded. We also took advantage of the opportunity to conduct a secondary exploratory analysis, by examining claims for TD (ICD-9 code of 333.85 and ICD-10 code of G24.0) including: (1) all patients with TD claims regardless of claims during the baseline period, (2) patients without TD claims during the baseline period, and (3) patients with TD claims during the baseline period who had at least one additional TD claim during the first year.

### Statistical analyses

Patient characteristics were compared between cases and controls using Wilcoxon signed-ranked tests for continuous variables and McNemar’s tests for categorical variables. Kaplan–Meier analyses with log-rank tests were used to estimate the median time to each outcome of interest between cases and controls. Univariable and multivariable Cox proportional hazard models were used to compare outcomes and the multivariable models adjusted for age (continuous), disease duration, CCI score, psychiatric comorbidity profile, psychotherapy use, and use of psychiatric medication. All variables evaluated at baseline were adjusted in the multivariable model for comprehensiveness, except for the variables matched or those which may cause collinearity. Hazard ratios (HRs) and corresponding 95% confidence intervals (CIs) were reported for the Cox proportional hazard models.

## Results

### Baseline characteristics

**Fig. 1:**
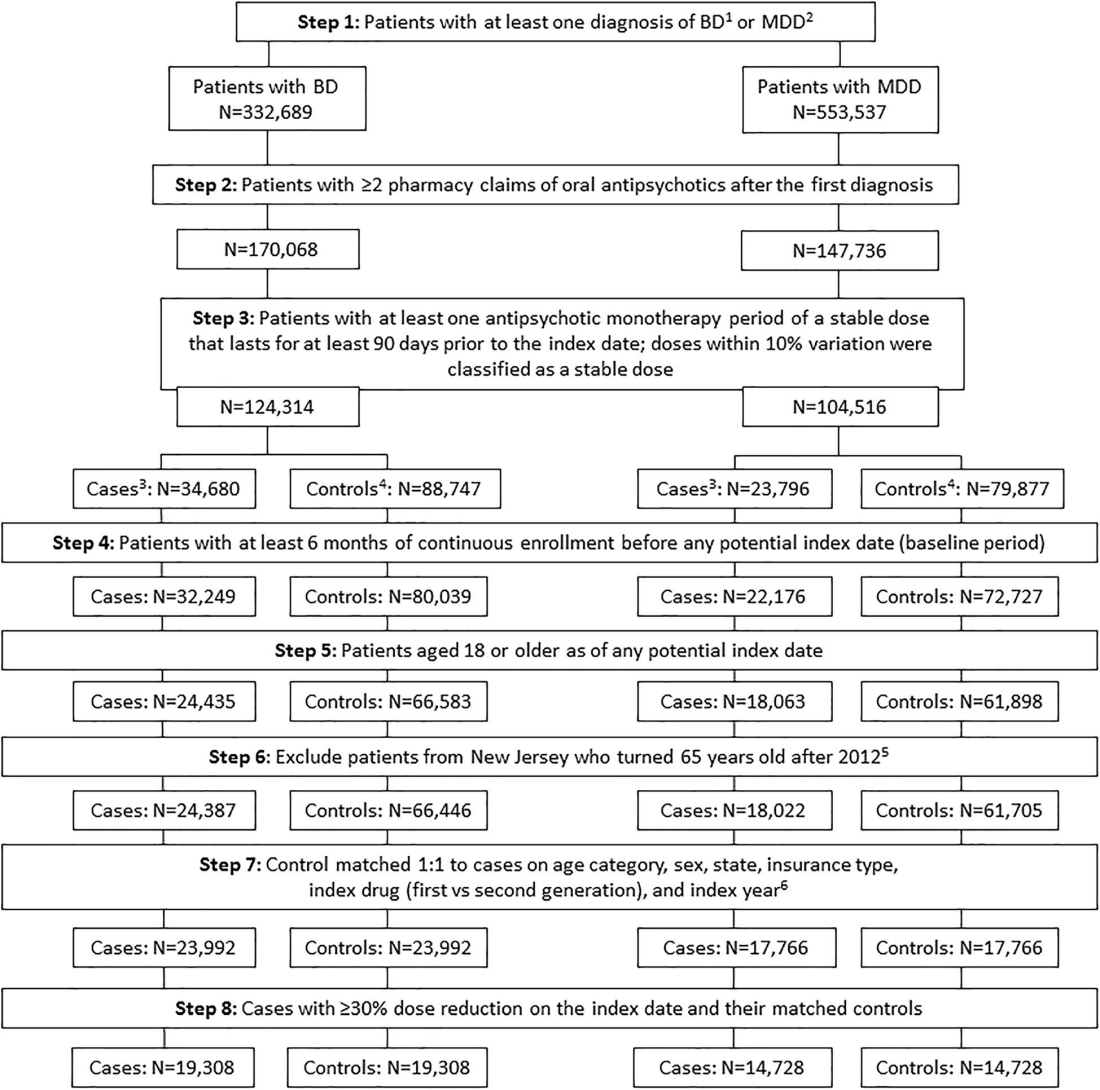
Sample selection for the BD and MDD groups. Patients were selected for case and control cohorts from a Medicaid claims database representing six US states and the most recent 6 years of data as detailed in Methods. BD: bipolar disorder; ICD-9/10: *International Classification of Diseases, 9th/10th Revision*; MDD: major depressive disorder. ^1^Diagnoses for BD were based on ICD-9 codes 296.0x, 296.1x, 296.4x, 296.5x, 296.6x, 296.7x, or 296.8x; and ICD-10 codes F30.x and F31.x from the Medicaid claims database (the most recent 6 years for data of each state). ^2^Diagnoses for MDD were based on ICD-9 codes 296.2x and 296.3x; and ICD-10 codes F32.x and F33.x from the Medicaid claims database (the most recent 6 years for data of each state). ^3^Cases were defined as patients at a stable monotherapy dose for a ≥ 90-day period and then experienced an **≥**10% dose reduction during the same monotherapy period. The first prescription date for the dose reduction fill was defined as a dose reduction starting date and was a potential index date. ^4^Controls were defined as patients who did not have a dose reduction and who had a stable dose monotherapy period that lasted for ≥91 days. The first prescription fill after the first 90 days of this stable dose monotherapy period was defined as a potential index date. ^5^Exclusion was based on dual eligibility for Medicare and Medicaid and the inability to capture drug claim information through Medicare claims. ^6^Cases were not included in the subsequent analysis if they could not be adequately matched on all of the matching characteristics, including: age, sex, type of health plan, state, index drug (first- vs second-generation antipsychotic), and index year

In the BD group, 23,992 patients were included in the ≥10% dose-reduction cohort and paired with an equal number of controls (Fig. 1). There were 19,308 patients who had a ≥ 30% antipsychotic dose reduction. For cases who experienced ≥10% dose reductions and their matched controls, the mean age was 41.0 years, 37.8% were men, and 42.7 and 20.1% had fee-for-service (FFS) and health maintenance organization (HMO) insurance plans, respectively (Table 1). The mean BD disease duration was significantly longer for cases (26.0 months) vs. controls (18.8 months), and the mean duration of follow-up was significantly longer for controls (6.5 months) vs. cases (4.0 months; both *P* <  0.001). Controls had significantly higher rates of substance use disorders, anxiety disorders, MDD, and other depressive disorders (all *P* <  0.001). Cases had higher rates of claims for schizophrenia than controls (*P* <  0.001). TD was present in 0.1% of both cases and controls. The CCI was similar for cases and controls (*P* = 0.64).

**Table 1:** Baseline Characteristics of Patients With ≥10% Antipsychotic Dose Reduction in the BD and MDD Groups

Demographics	BD			MDD		
	Case	Control	*P*-value	Case	Control	*P*-value
	N = 23,992	N = 23,992		N = 17,766	N = 17,766	
**Age, mean ± SD, years**	41.00 ± 13.83	41.01 ± 13.82	0.27	44.18 ± 14.15	44.17 ± 14.14	0.51
**Men, n (%)**	9076 (37.83%)	9076 (37.83%)	–	5958 (33.54%)	5958 (33.54%)	–
**State, n (%)**			–			–
Iowa	1831 (7.63%)	1831 (7.63%)		863 (4.86%)	863 (4.86%)	
Kansas	1808 (7.54%)	1808 (7.54%)		1243 (7.00%)	1243 (7.00%)	
Mississippi	1432 (5.97%)	1432 (5.97%)		1243 (7.00%)	1243 (7.00%)	
Missouri	10,297 (42.92%)	10,297 (42.92%)		8796 (49.51%)	8796 (49.51%)	
New Jersey	5594 (23.32%)	5594 (23.32%)		3805 (21.42%)	3805 (21.42%)	
Wisconsin	3030 (12.63%)	3030 (12.63%)		1816 (10.22%)	1816 (10.22%)	
**Insurance type,*n*(%)**			–			–
FFS	10,255 (42.74%)	10,255 (42.74%)		7513 (42.29%)	7513 (42.29%)	
HMO	4817 (20.08%)	4817 (20.08%)		3219 (18.12%)	3219 (18.12%)	
Mixed	8920 (37.18%)	8920 (37.18%)		7034 (39.59%)	7034 (39.59%)	
**Disease duration, mean ± SD, months**	26.00 ± 17.49	18.80 ± 16.65	< 0.001*	25.15 ± 17.22	18.00 ± 16.08	< 0.001*
**Duration of follow-up, mean ± SD, months**	3.94 ± 6.53	6.46 ± 9.19	< 0.001*	4.21 ± 6.77	6.37 ± 8.83	< 0.001*
**Index characteristics,*n*(%)**						
*Index Year*			–			–
2008	349 (1.45%)	349 (1.45%)		233 (1.31%)	233 (1.31%)	
2009	1342 (5.59%)	1342 (5.59%)		790 (4.45%)	790 (4.45%)	
2010	1908 (7.95%)	1908 (7.95%)		1254 (7.06%)	1254 (7.06%)	
2011	2967 (12.37%)	2967 (12.37%)		1860 (10.47%)	1860 (10.47%)	
2012	4014 (16.73%)	4014 (16.73%)		2803 (15.78%)	2803 (15.78%)	
2013	4203 (17.52%)	4203 (17.52%)		3077 (17.32%)	3077 (17.32%)	
2014	2877 (11.99%)	2877 (11.99%)		2148 (12.09%)	2148 (12.09%)	
2015	3157 (13.16%)	3157 (13.16%)		2447 (13.77%)	2447 (13.77%)	
2016	2618 (10.91%)	2618 (10.91%)		2545 (14.33%)	2545 (14.33%)	
2017	557 (2.32%)	557 (2.32%)		609 (3.43%)	609 (3.43%)	
*Index Drug Class*			–			–
First-generation antipsychotic	1421 (5.92%)	1421 (5.92%)		1032 (5.81%)	1032 (5.81%)	
Second-generation antipsychotic	22,571 (94.08%)	22,571 (94.08%)		16,734 (94.19%)	16,734 (94.19%)	
**CCI, mean ± SD**	0.61 ± 1.19	0.60 ± 1.17	0.64	0.76 ± 1.32	0.74 ± 1.33	0.26
**Psychiatric comorbidities,*n*(%)**						
Substance-related and addictive disorders	6539 (27.25%)	6952 (28.98%)	< 0.001*	4706 (26.49%)	4865 (27.38%)	0.05
Anxiety disorders	5630 (23.47%)	6129 (25.55%)	< 0.001*	4879 (27.46%)	5351 (30.12%)	< 0.001*
BD	–	–	–	5190 (29.21%)	4654 (26.20%)	< 0.001*
Bipolar-related disorders (excluding BD)	210 (0.88%)	226 (0.94%)	0.47	178 (1.00%)	192 (1.08%)	0.50
MDD	4505 (18.78%)	5081 (21.18%)	< 0.001*	–	–	–
Depressive disorders (excluding MDD)	4429 (18.46%)	4950 (20.63%)	< 0.001*	4226 (23.79%)	4678 (26.33%)	< 0.001*
Personality disorders	1322 (5.51%)	1234 (5.14%)	0.08	1025 (5.77%)	884 (4.98%)	< 0.001*
Schizophrenia	5783 (24.10%)	5139 (21.42%)	< 0.001*	3819 (21.50%)	3108 (17.49%)	< 0.001*
Schizophrenia spectrum disorders	1961 (8.17%)	1952 (8.14%)	0.89	1576 (8.87%)	1517 (8.54%)	0.27
Sleep-wake disorders	2395 (9.98%)	2453 (10.22%)	0.38	2044 (11.51%)	2142 (12.06%)	0.11
Trauma- and stressor-related disorders	2939 (12.25%)	2985 (12.44%)	0.52	2559 (14.40%)	2636 (14.84%)	0.24
Tardive dyskinesia	18 (0.08%)	26 (0.11%)	0.29	19 (0.11%)	17 (0.10%)	0.87
**Non-psychiatric comorbidities,*n*(%)**						
AIDS/HIV	277 (1.15%)	280 (1.17%)	0.93	242 (1.36%)	259 (1.46%)	0.47
Cancer	412 (1.72%)	463 (1.93%)	0.09	416 (2.34%)	444 (2.50%)	0.35
Cerebrovascular disease	809 (3.37%)	744 (3.10%)	0.09	881 (4.96%)	812 (4.57%)	0.08
Congestive heart failure	729 (3.04%)	689 (2.87%)	0.28	734 (4.13%)	773 (4.35%)	0.31
Chronic pulmonary disease	5305 (22.11%)	5309 (22.13%)	0.97	4362 (24.55%)	4240 (23.87%)	0.13
Dementia	352 (1.47%)	275 (1.15%)	< 0.01*	456 (2.57%)	357 (2.01%)	< 0.001*
Diabetes with chronic complication	816 (3.40%)	792 (3.30%)	0.55	800 (4.50%)	787 (4.43%)	0.76
Diabetes without chronic complication	3259 (13.58%)	2985 (12.44%)	< 0.001*	2741 (15.43%)	2590 (14.58%)	< 0.05*
Hemiplegia or paraplegia	289 (1.20%)	236 (0.98%)	< 0.05*	291 (1.64%)	243 (1.37%)	< 0.05*
Mild liver disease	1125 (4.69%)	1106 (4.61%)	0.69	954 (5.37%)	934 (5.26%)	0.65
Metastatic solid tumor	60 (0.25%)	79 (0.33%)	0.13	70 (0.39%)	83 (0.47%)	0.32
Myocardial infarction	230 (0.96%)	242 (1.01%)	0.61	195 (1.10%)	205 (1.15%)	0.65
Moderate or severe liver disease	87 (0.36%)	77 (0.32%)	0.48	76 (0.43%)	76 (0.43%)	1.00
Peptic ulcer disease	157 (0.65%)	170 (0.71%)	0.51	147 (0.83%)	144 (0.81%)	0.91
Peripheral vascular disease	811 (3.38%)	666 (2.78%)	< 0.001*	767 (4.32%)	716 (4.03%)	0.17
Renal disease	545 (2.27%)	497 (2.07%)	0.14	520 (2.93%)	490 (2.76%)	0.35
Rheumatic disease	336 (1.40%)	333 (1.39%)	0.94	352 (1.98%)	374 (2.11%)	0.43
**Psychotherapy,*n*(%)**						
Psychoanalysis	1 (0.00%)	2 (0.01%)	1.00	0 (0.00%)	0 (0.00%)	–
Psychotherapy in crisis	46 (0.19%)	44 (0.18%)	0.92	42 (0.24%)	39 (0.22%)	0.82
Psychotherapy non-crisis	3806 (15.86%)	4039 (16.83%)	< 0.01*	3249 (18.29%)	3463 (19.49%)	< 0.01*
**Psychiatric medication,*n*(%)**						
Antidepressant	13,490 (56.23%)	13,776 (57.42%)	< 0.001*	11,906 (67.02%)	12,036 (67.75%)	< 0.05*
Anticholinergic	2649 (11.04%)	2043 (8.52%)	< 0.001*	1736 (9.77%)	1232 (6.93%)	< 0.001*
Sedative	3494 (14.56%)	3473 (14.48%)	0.47	2906 (16.36%)	2878 (16.20%)	0.68
Mood stabilizer	10,302 (42.94%)	9507 (39.63%)	< 0.001*	6574 (37.00%)	6171 (34.73%)	< 0.001*
Anxiety medication	8015 (33.41%)	8446 (35.20%)	< 0.001*	6607 (37.19%)	6869 (38.66%)	< 0.01*
ADHD medication	2053 (8.56%)	1995 (8.32%)	0.31	1100 (6.19%)	1106 (6.23%)	0.91

*ADHD* attention deficit hyperactivity disorder, *AIDS/HIV* acquired immune deficiency syndrome/human immunodeficiency virus infection, *BD* bipolar disorder, *CCI* Charlson Comorbidity Index, *FFS* fee-for-service, *HMO* health maintenance organization, *MDD* major depressive disorder, *SD* standard deviation. *P < 0.05

In the MDD group, 17,766 patients were included in the > 10% dose-reduction cohort and paired with an equal number of controls. There were 14,728 patients who had a ≥ 30% antipsychotic dose reduction (Fig. 1). For MDD cases who experienced ≥10% dose reductions and their matched controls, the mean age was 44.2 years, 33.5% were men, and 42.3 and 18.1% had FFS and HMO insurance, respectively (Table 1). The mean disease duration was significantly longer for MDD cases (25.2 months) vs. controls (18.0 months), and the mean duration of follow-up was significantly longer for MDD controls (6.4 months) vs. cases (4.2 months; both *P* <  0.001). Controls had significantly higher rates of substance use disorders, anxiety disorders, and depressive disorders other than MDD (both *P* <  0.001) compared with cases. Conversely, cases had significantly higher rates of claims for BD, personality disorders, and schizophrenia (all *P* <  0.001) than controls. TD was present in 0.1% of both cases and controls. The CCI was similar for MDD cases and controls (*P* = 0.26). Patient characteristics among patients with a ≥ 30% dose reduction were similar to patients with a ≥ 10% dose reduction in both BD and MDD groups (Additional file 2).

### Dosing patterns

The most commonly used antipsychotic drugs among BD and MDD cases and controls were similar (Additional file 3). The mean dosages of all antipsychotic medications at the index date were higher among cases than controls in both diagnostic groups. Across all antipsychotic medications, 25% of patients had a dose reduction of approximately 33% on the index date, and approximately half of patients had a dose reduction of 50% or less; the mean dose reductions ranged from 45.1% (ziprasidone; MDD group) to 56.6% (paliperidone; BD group) (Additional file 4).

### Hospital utilization outcomes

**Fig. 2:**
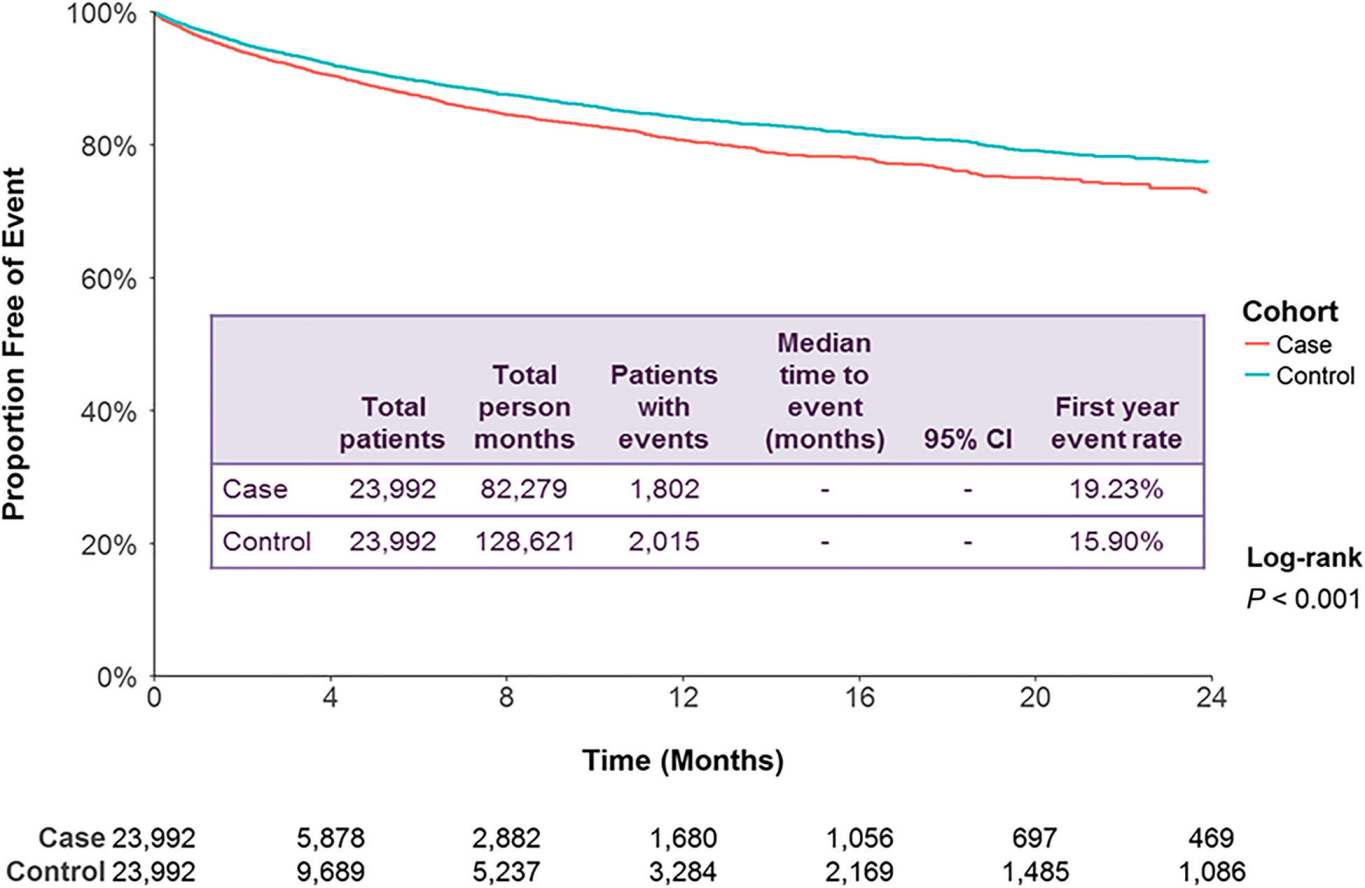
Patient claims analyzed for BD-related inpatient admissions after ≥10% dose reductions of antipsychotic medication. Outcomes for case and control cohorts were assessed using Kaplan–Meier analysis and compared using a log-rank test. The number of patients at risk is represented for each time point. Case and control cohorts for ≥10%, *N* = 23,992 each. CI: confidence interval; BD: bipolar disorder

BD cases with a ≥ 10% dose reduction had a higher rate of a BD-related admission compared to controls (*P* <  0.001; Fig. 2). The first-year event rate for BD-related admission was 19.2% for cases and 15.9% for controls, a difference of 3.3%. The adjusted HR was 1.22 (95% CI: 1.15, 1.31; *P* <  0.001; Table 2). The first-year event rate of psychiatric admissions for other disorders was 36.0% for cases and 32.1% for controls, a difference of 3.9% (*P* <  0.001; Additional file 5). The adjusted HR was 1.19 (95% CI: 1.13, 1.24; *P* <  0.001; Table 2).

**Table 2:** Comparisons of Inpatient and Emergency Room Admissions and TD Claims Among Patients With ≥10% or ≥ 30% Antipsychotic Dose Reductions vs Controls in the BD and MDD Groups

	Adjusted HR (95% CI)							
	BD				MDD			
	≥10%	***P***	≥30%	***P***	≥10%	***P***	≥30%	***P***
Disease-specific admission	1.22 (1.15, 1.31)	< 0.001*	1.24 (1.15, 1.33)	< 0.001*	1.22 (1.11, 1.34)	< 0.001*	1.27 (1.14, 1.41)	< 0.001*
Psychiatric admission	1.19 (1.13, 1.24)	< 0.001*	1.19 (1.13, 1.25)	< 0.001*	1.17 (1.11, 1.23)	< 0.001*	1.16 (1.10, 1.24)	< 0.001*
All-cause IP admission	1.17 (1.12, 1.23)	< 0.001*	1.17 (1.11, 1.24)	< 0.001*	1.11 (1.05, 1.16)	< 0.001*	1.12 (1.06, 1.18)	< 0.001*
All-cause ER visit	1.09 (1.05, 1.13)	< 0.001*	1.10 (1.06, 1.15)	< 0.001*	1.07 (1.02, 1.11)	< 0.01*	1.08 (1.03, 1.13)	< 0.01*
TD claim for all patients	1.45 (0.75, 2.82)	0.27	1.83 (0.87, 3.88)	0.11	2.40 (1.19, 4.83)	0.01*	2.51 (1.14, 5.52)	0.02*
TD claim for patients without baseline TD	1.95 (0.90, 4.22)	0.09	2.11 (0.88, 5.07)	0.09	2.34 (1.05, 5.21)	0.04*	2.90 (1.15, 7.29)	0.02*

*BD* bipolar disorder, *CI* confidence interval, *ER* emergency room, *HR* hazard ratio, *IP* inpatient, *MDD* major depressive disorder, *TD* tardive dyskinesia. *P < 0.05

BD cases had a higher all-cause inpatient admission event rate (36.3%) than their matched controls (32.7%; Additional file 6) with an adjusted HR of 1.17 (95% CI: 1.12, 1.23; *P* <  0.001; Table 2). BD cases had a higher first-year ER visit event rate (52.1%) than matched controls (49.9%; Additional file 7) with an adjusted HR of 1.09 (95% CI: 1.05, 1.13, *P* <  0.001; Table 2). Results were similar for BD cases with a ≥ 30% dose reduction vs. controls.

**Fig. 3:**
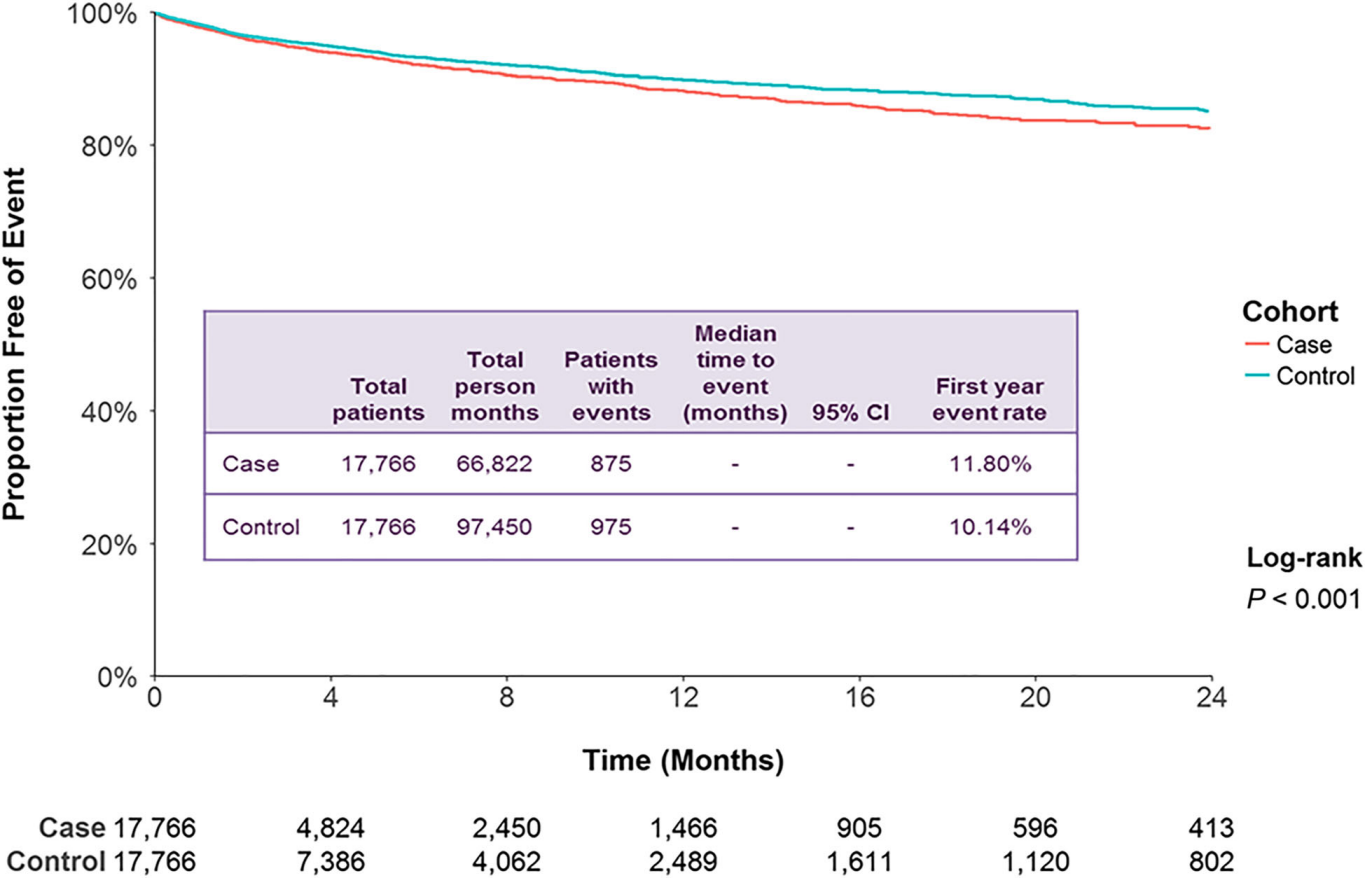
Patient claims analyzed for MDD-related inpatient admissions after ≥10% dose reductions of antipsychotic medication. Outcomes for case and control cohorts were assessed using Kaplan–Meier analysis and compared using a log-rank test. The number of patients at risk is represented for each time point. Case and control cohorts for ≥10%, *N* = 17,766 each. CI: confidence interval; MDD: major depressive disorder

MDD cases with a ≥ 10% dose reduction had a higher rate of an MDD-related admission compared to controls (*P* <  0.001; Fig. 3). The first-year event rate for MDD-related admission was 11.8% for cases and 10.1% for controls, a difference of 1.7%. The adjusted HR was 1.22 (95% CI: 1.11, 1.34; *P* <  0.001; Table 2). MDD cases (32.5%) had a higher rate of psychiatric admissions for other disorders compared to controls (30.1%; *P* <  0.001; Additional file 8) with an adjusted HR of 1.17 (95% CI: 1.11, 1.23, *P* <  0.001; Table 2).

MDD cases had a higher all-cause inpatient admission event rate (37.6%) than their matched controls (35.7%; Additional file 9). The adjusted HR was 1.11 (95% CI: 1.05, 1.16; *P* <  0.001; Table 2). MDD cases also had a higher first-year ER visit event rate (51.1%) than matched controls (50.2%; Additional file 10). The adjusted HR was 1.07 (95% CI: 1.02, 1.11; *P* <  0.001; Table 2). Results were similar for MDD cases with a ≥ 30% dose reduction vs. controls.

### TD claims

The overall difference in TD claims between BD cases and controls was not significant: the adjusted HR was 1.45 (95% CI: 0.75, 2.82; *P* = 0.27) for the ≥10% dose-reduction analysis and 1.83 (95% CI: 0.87, 3.88; *P* = 0.11) for the ≥30% dose-reduction analysis (Table 2).

When excluding patients with TD claims at baseline, the adjusted HR of a new TD claim was 1.95 (95% CI: 0.90, 4.22; *P* = 0.09) for the ≥10% dose-reduction analysis and 2.11 (95% CI: 0.88, 5.07; *P* = 0.09) for the ≥30% dose-reduction analysis (Table 2). Among patients with a pre-existing TD claim during the baseline period, the percentages of patients having at least one additional TD claim during the first year of the study period were compared between the dose-reduction cohort and the control cohort in both the ≥10% (16/18 [88.9%] vs 23/26 [88.5%], *P* = 0.97) and the ≥30% dose-reduction analyses (14/15 [93.3%] vs 20/23 [87.0%]; *P* = 0.53).

MDD cases with dose reduction had a higher risk of TD claims vs. controls: the adjusted HR was 2.40 (95% CI: 1.19, 4.83; *P* = 0.01) for the 10% dose reduction analysis and 2.51 (95% CI: 1.14, 5.52; *P* = 0.02) for the ≥30% dose-reduction analysis (Table 2). When excluding patients with TD claims during baseline, the adjusted HR of a new TD claim was 2.34 (95% CI: 1.05, 5.21; *P* = 0.04) for the ≥10% dose-reduction analysis and 2.90 (95% CI: 1.15, 7.29; *P* = 0.02) for the ≥30% dose reduction analysis (Table 2). Among patients with a pre-existing TD claim during the baseline period, the percentages of patients having at least one additional TD claim during the first year of the study period were compared between the dose-reduction cohort and the control cohort in both the ≥10% (16/19 [84.2%] vs 15/17 [88.2%], *P* = 0.73) and the ≥30% dose-reduction analyses (12/13 [92.3%] vs 14/16 [87.5%]; *P* = 0.67).

## Discussion

While the use of antipsychotics in BD and MDD has become commonplace, there is a need for long-term evidence on the efficacy, safety, and necessity of maintenance antipsychotics in these populations. Although the accepted role of antipsychotics started during acute mania may be reflected in the greater number of patients with BD remaining on antipsychotics in the study sample, the necessity of antipsychotics in the maintenance treatment of BD and MDD is less well established [13]. The risk of TD and other side effects with prolonged treatment is substantial. This study used real-world claims data to compare the risk of both all-cause and mental health-related admissions and ER visits in patients with BD or MDD who had dose reductions with matched controls on stable oral dosages of antipsychotics. The results show that among patients with mood disorders, dose reductions resulted in small but statistically significant increases in inpatient admissions and ER visits, similar to previous findings among patients with schizophrenia [23].

As was observed in schizophrenia patients [23], differences in first-year hospital event rates were slight, especially between MDD cases and controls, but were statistically significant given the large sample size. The clinical meaningfulness of these differences should be considered by healthcare decision makers because even a 1% difference in event rates reflected an additional 240 patients using hospital services. Hospital re-admissions contribute to the economic burden for both patients and health systems [27]. An estimated 33.5 to 65.2% of the overall costs of treating patients with BD and approximately 38% of the cost of treating patients with MDD are attributable to patient hospitalization [28–30]. Comparing findings between mood disorder patients and schizophrenia patients in our previous analysis [23], BD patients were similar in disease-specific hospitalizations to patients with schizophrenia following dose reductions, but both of these disorders resulted in more hospitalizations than MDD patients, reflecting perhaps the greater heterogeneity in severity of the MDD subtype. TD claims were twice as common in schizophrenia which is consistent with recent reports [8, 10–12, 18, 23], but too few to reach definitive conclusions.

Analysis of the effects of dose reduction on claims for TD were only exploratory, descriptive and secondary to the primary outcome objective of hospitalizations. Moreover, interpretation of the relationship between dose reduction and TD is limited in this study by the shorter follow-up period in cases and the very low frequency of TD claims suggesting a high rate of false negative diagnoses [31]. In fact, the prevalence rate of TD reported in retrospective database studies like ours is significantly less than in prospective clinical trials of mood disorder patients [11, 12, 23, 32–34]. These results clearly indicate that TD is seriously underreported in claims databases [34] and should be documented in a more systematic fashion. Nevertheless, our preliminary exploration found no evidence of fewer claims for TD after dose reduction. In fact, there were more claims in MDD patients following dose reduction, possibly representing withdrawal dyskinesias. Both MDD and BD patients with dose reduction also had higher rates of anticholinergic treatment; these drugs exacerbate symptoms of TD, which may then become apparent after antipsychotic dose reduction. In addition, it is possible that physicians proactively reduced the dose of antipsychotics due to some early signs of TD. Finally, apart from dose reduction, alternative strategies for treating TD may include antipsychotic discontinuation or switching to less-potent antipsychotics, or, more recently, to approved vesicular monoamine transporter-2 inhibitors (deutetrabenazine, valbenazine).

The strengths of this study include the use of a large claims data set based on real-world practice settings. Claims were analyzed from multiple states, with matched case and control groups. There are several limitations to the analyses. The reasons for antipsychotic dose reductions and whether dose reduction affected outcomes positively in other clinical domains were not assessed. For example, patients in the dose-reduction group had higher mean dosages in the pre-index period, as well as more cases of diabetes and use of anticholinergics, presumably for acute extrapyramidal symptoms, which may have prompted dose reductions [31]. Other changes in mood stabilizers or antidepressants could have influenced the results. A small number of cases and controls in both the BD (5.8% vs. 5.6%) and MDD (4.4% vs 4.1%) groups received a long-acting injectable antipsychotic in addition to their oral antipsychotic, but these differences were not significant (chi-square, *P*-value > 0.05). Dose-reduction groups may have had more hospitalizations because they were more severely ill. However, patients with more severe disease would have been less likely to have doses reduced, and more severely ill patients were excluded if they received multiple oral antipsychotics during the baseline period or had recent increases in doses. Cases of BD and MDD were more likely to receive diagnoses of schizophrenia whereas other comorbid diagnoses (e.g., substance use disorder) that contribute to hospitalization were more common among controls. Both cohorts seemed to have patients with multiple psychiatric diagnoses as documented in coded medical records, which is typical of real-world studies and can reflect diagnostic uncertainties, psychiatric comorbidities, or the difficulty of determining which symptoms (e.g., psychotic episodes, depressed mood) are predominant. Outcome measures were also controlled for covariates in the Cox model that reflect severity.

Nonadherence to treatment is another factor that may increase the likelihood of hospitalization, but even if nonadherence contributed to hospitalizations, that would still be evidence suggesting untoward effects of dose reduction. High attrition during the study and the difference between cases and controls were due to the differences in outcomes. Kaplan–Meier analyses and Cox models were used to handle the attrition and censoring of patients; these statistical tools are designed to account for censored or truncated time-to-event data [35].

Finally, these results were limited to patients with mood disorders who received extended treatment with antipsychotics, and therefore are not generalizable to the much larger population of patients with mood disorders who respond to treatment without the need for antipsychotics.

## Conclusions

These results showing small but statistically significant effects of antipsychotic dose reductions on hospitalization rates, especially among patients with BD, reinforce similar findings in patients with schizophrenia [23]. The results did not support dose reduction for preventing or treating TD, but definitive conclusions could not be reached because of possible withdrawal dyskinesias, the limited duration of follow-up, use of anticholinergics, and underreporting of the diagnosis. Therefore, decisions on dose reduction of antipsychotics in patients with severe, psychotic, or recurrent mood disorders who require maintenance antipsychotic treatment should be carefully considered on an individualized basis. These results highlight the need for long-term studies of the necessity and safety of maintenance antipsychotic treatment in patients with mood disorders, and of alternative management strategies to address side effects that emerge during treatment.

## Supplementary information

**Additional file 1.** Diagnostic Codes for BD, MDD, and Other Psychiatric Conditions.

**Additional file 2.** Baseline Characteristics of Patients With ≥30% Antipsychotic Dose Reduction in the BD and MDD Groups.

**Additional file 3.** Dose Distributions Among Patients in the BD and MDD Groups During the ≤90-day Stable Dose Period Prior to the Index Date for the 10 Most Commonly Used Antipsychotic Medications.

**Additional file 4.** Dose Reduction Percentiles for the 10 Most Commonly Used Antipsychotic Medications Among Patients in the BD and MDD Groups.

**Additional file 5. **Psychiatric Admission With ≥10% Antipsychotic Dose Reduction in the BD Group. Patient claims were analyzed for psychiatric admissions related to BD for ≥10% dose reductions of antipsychotic medication. Outcomes for case and control cohorts were assessed using Kaplan–Meier analysis and compared using a log-rank test. The number of patients at risk is represented for each time point. Case and control cohorts for ≥10%, *N* = 23,992 each. BD: bipolar disorder; CI: confidence interval.

**Additional file 6. **All-Cause Inpatient Admissions Among Patients With ≥10% Antipsychotic Dose Reduction in the BD Group. Patient claims were analyzed for all-cause inpatient admissions related to BD for ≥10% dose reductions of antipsychotic medication. Outcomes for case and control cohorts were assessed using Kaplan–Meier analysis and compared using a log-rank test. The number of patients at risk is represented for each time point. Case and control cohorts for ≥10%, *N* = 23,992 each. BD: bipolar disorder; CI: confidence interval; IP: inpatient.

**Additional file 7. **All-Cause Emergency Room Visits Among Patients With ≥10% Antipsychotic Dose Reduction in the BD Group. Patient claims were analyzed for all-cause emergency room admissions related to BD for ≥10% dose reductions of antipsychotic medication. Outcomes for case and control cohorts were assessed using Kaplan–Meier analysis and compared using a log-rank test. The number of patients at risk is represented for each time point. Case and control cohorts for ≥10%, *N* = 23,992 each. BD: bipolar disorder; CI: confidence interval; ER: emergency room.

**Additional file 8. **Psychiatric Admission Among Patients With ≥10% Antipsychotic Dose Reduction in the MDD Group. Patient claims were analyzed for psychiatric admissions related to MDD for ≥10% dose reductions of antipsychotic medication. Outcomes for case and control cohorts were assessed using Kaplan–Meier analysis and compared using a log-rank test. The number of patients at risk is represented for each time point. Case and control cohorts for ≥10%, *N* = 17,766 each. CI: confidence interval; MDD: major depressive disorder.

**Additional file 9. **All-Cause inpatient Admissions Among Patients With ≥10% Antipsychotic Dose Reduction in the MDD Group. Patient claims were analyzed for all-cause inpatient admissions related to MDD for ≥10% dose reductions of antipsychotic medication. Outcomes for case and control cohorts were assessed using Kaplan–Meier analysis and compared using a log-rank test. The number of patients at risk is represented for each time point. Case and control cohorts for ≥10%, *N* = 17,766 each. CI: confidence interval; IP: inpatient; MDD: major depressive disorder.

**Additional file 10. **All-Cause ER Visits Among Patients With ≥10% Antipsychotic Dose Reduction in the MDD Group. Patient claims were analyzed for all-cause emergency room admissions related to MDD for ≥10% dose reductions of antipsychotic medication. Outcomes for case and control cohorts were assessed using Kaplan–Meier analysis and compared using a log-rank test. The number of patients at risk is represented for each time point. Case and control cohorts for ≥10%, *N* = 17,766 each. CI: confidence interval; ER: emergency room; MDD: major depressive disorder.

## Data Availability

Restrictions apply to the availability of these data, which were used under license for the current study, and are not publicly available. The data are available from the authors upon reasonable request and with permission of Analysis Group, Inc.

## Abbreviations

BD: Bipolar disorder
CCI: Charlson Comorbidity Index
CI: Confidence interval
ER: Emergency room
FGA: First-generation antipsychotics
FFS: Fee-for-service
HR: Hazard ratio
HMO: Health maintenance organization
MDD: Major depressive disorder
SD: Standard deviation
SGA: Second-generation antipsychotics
TD: Tardive dyskinesia
