# Supplementary material for: Hospital utilization rates following antipsychotic dose reduction in mood disorders: implications for treatment of tardive dyskinesia
Source: BMC Psychiatry. 2020 Jul 11;20:365. doi: 10.1186/s12888-020-02748-0 (PMC7353680; doi:10.1186/s12888-020-02748-0)
Supplement: Supplementary file 1 — Additional file 1. Diagnostic Codes for BD, MDD, and Other Psychiatric Conditions. [file 12888_2020_2748_MOESM1_ESM.docx]

**Additional File 1. Diagnostic Codes for BD, MDD, and Other Psychiatric Conditions.**

| Condition | ICD-9 Code | ICD-10 Code |
| --- | --- | --- |
| BD | 296.0, 296.1, 296.4, 296.5, 296.6, 296.7, 296.8 | F30, F31 |
| MDD | 296.2, 296.3 | F32, F33 |
| Bipolar-related disorders (excluding BD) | 293.83, 301.13 | F0630, F340 |
| Depressive disorders (excluding MDD) | 300.4, 625.4, 311 | F341, N943 |
| Schizophrenia | 295.xx | F20, F25 |
| Schizophrenia-spectrum and other psychotic disorders (excluding schizophrenia) | 293.81, 293.82, 293.89, 297.1, 298.8, 298.9, 301.22 | F062, F060, F061, F53, F22, F23, F29, F21 |
| Substance-related and addictive disorders | 291.81, 291.9, 292.0, 292.89, 292.9, 303.00, 303.90, 304.00, 304.10, 304.20, 304.30, 304.40, 304.50, 304.60, 304.90, 305.00, 305.1, 305.20, 305.30, 305.40, 305.50, 305.60, 305.70, 305.90 | F10, F11, F12, F13, F14, F15, F17, F18, F19 |
| Trauma- and stressor-related disorders | 308.3, 309.81, 309.0, 309.24, 309.28, 309.3, 309.4, 309.9, 309.89, 313.89 | F43, F938, F941, F988 |
| Anxiety disorders | 309.21, 312.23, 300.29, 300.23, 300.01, 300.22, 300.02, 293.84, 300.09, 300.00 | F930, F91, F40, F41, F064 |
| Sleep–wake disorders | 307.45, 307.46, 307.47, 327.21, 327.23, 327.24, 327.25, 327.26, 327.42, 333.94, 347.00, 347.01, 347.10, 780.52, 780.54, 780.57, 780.59, 786.04 | F51, F950, G47, G2581, R063 |
| Personality disorders | 301.0, 301.1, 301.20, 301.4, 301.5, 301.6, 301.81, 301.82, 301.83, 301.89, 301.9 | F60 |

BD: bipolar disorder, ICD: International Classification of Diseases, MDD: major depressive disorder.
