# Supplementary material for: Hospital utilization rates following antipsychotic dose reduction in mood disorders: implications for treatment of tardive dyskinesia
Source: BMC Psychiatry. 2020 Jul 11;20:365. doi: 10.1186/s12888-020-02748-0 (PMC7353680; doi:10.1186/s12888-020-02748-0)
Supplement: Supplementary file 2 — Additional file 2. Baseline Characteristics of Patients With ≥30% Antipsychotic Dose Reduction in the BD and MDD Groups. [file 12888_2020_2748_MOESM2_ESM.docx]

**Additional File 2. Baseline Characteristics of Patients With ≥30% Antipsychotic Dose Reduction in the BD and MDD Groups.**

| **Demographics** | **BD** | | |  | **MDD** | | |
| --- | --- | --- | --- | --- | --- | --- | --- |
|  | **Case** | **Control** | ***P*-value** |  | **Case** | **Control** | ***P*-value** |
|  | **N = 19308** | **N = 19308** |  |  | **N = 14728** | **N = 14728** |  |
| **Age, mean ± SD, years** | 40.98 ± 13.80 | 40.98 ± 13.80 | 0.51 |  | 44.23 ± 14.14 | 44.22 ± 14.13 | 0.69 |
| **Men, n (%)** | 7181 (37.19%) | 7181 (37.19%) | - |  | 4880 (33.13%) | 4880 (33.13%) | - |
| **State, n (%)** |  |  | - |  |  |  | - |
| Iowa | 1517 (7.86%) | 1517 (7.86%) |  |  | 725 (4.92%) | 725 (4.92%) |  |
| Kansas | 1466 (7.59%) | 1466 (7.59%) |  |  | 1032 (7.01%) | 1032 (7.01%) |  |
| Mississippi | 1186 (6.14%) | 1186 (6.14%) |  |  | 1055 (7.16%) | 1055 (7.16%) |  |
| Missouri | 8346 (43.23%) | 8346 (43.23%) |  |  | 7325 (49.74%) | 7325 (49.74%) |  |
| New Jersey | 4431 (22.95%) | 4431 (22.95%) |  |  | 3161 (21.46%) | 3161 (21.46%) |  |
| Wisconsin | 2362 (12.23%) | 2362 (12.23%) |  |  | 1430 (9.71%) | 1430 (9.71%) |  |
| **Insurance type, n (%)** |  |  | - |  |  |  | - |
| FFS | 8263 (42.80%) | 8263 (42.80%) |  |  | 6261 (42.51%) | 6261 (42.51%) |  |
| HMO | 3829 (19.83%) | 3829 (19.83%) |  |  | 2620 (17.79%) | 2620 (17.79%) |  |
| Mixed | 7216 (37.37%) | 7216 (37.37%) |  |  | 5847 (39.70%) | 5847 (39.70%) |  |
| **Disease duration, mean ± SD, months** | 25.90 ± 17.53 | 18.82 ± 16.69 | <0.001* |  | 25.05 ± 17.24 | 18.01 ± 16.11 | <0.001* |
| **Duration of follow-up, mean ± SD, months** | 3.63 ± 6.12 | 6.46 ± 9.24 | <0.001* |  | 4.00 ± 6.50 | 6.34 ± 8.81 | <0.001* |
| **Index characteristics, n (%)** |  |  |  |  |  |  |  |
| *Index Year* |  |  | - |  |  |  | - |
| 2008 | 288 (1.49%) | 288 (1.49%) |  |  | 195 (1.32%) | 195 (1.32%) |  |
| 2009 | 1029 (5.33%) | 1029 (5.33%) |  |  | 652 (4.43%) | 652 (4.43%) |  |
| 2010 | 1493 (7.73%) | 1493 (7.73%) |  |  | 1017 (6.91%) | 1017 (6.91%) |  |
| 2011 | 2339 (12.11%) | 2339 (12.11%) |  |  | 1506 (10.23%) | 1506 (10.23%) |  |
| 2012 | 3270 (16.94%) | 3270 (16.94%) |  |  | 2343 (15.91%) | 2343 (15.91%) |  |
| 2013 | 3394 (17.58%) | 3394 (17.58%) |  |  | 2571 (17.46%) | 2571 (17.46%) |  |
| 2014 | 2370 (12.27%) | 2370 (12.27%) |  |  | 1781 (12.09%) | 1781 (12.09%) |  |
| 2015 | 2548 (13.20%) | 2548 (13.20%) |  |  | 2030 (13.78%) | 2030 (13.78%) |  |
| 2016 | 2108 (10.92%) | 2108 (10.92%) |  |  | 2110 (14.33%) | 2110 (14.33%) |  |
| 2017 | 469 (2.43%) | 469 (2.43%) |  |  | 523 (3.55%) | 523 (3.55%) |  |
| *Index Drug Class* |  |  | - |  |  |  | - |
| First-generation antipsychotic | 1151 (5.96%) | 1151 (5.96%) |  |  | 832 (5.65%) | 832 (5.65%) |  |
| Second-generation antipsychotic | 18157 (94.04%) | 18157 (94.04%) |  |  | 13896 (94.35%) | 13896 (94.35%) |  |
|  |  |  |  |  |  |  |  |
| **CCI, mean ± SD** | 0.62 ± 1.20 | 0.60 ± 1.16 | 0.25 |  | 0.77 ± 1.33 | 0.75 ± 1.33 | 0.37 |
| **Psychiatric comorbidities N (%)** |  |  |  |  |  |  |  |
| Substance-related and addictive disorders | 5339 (27.65%) | 5591 (28.96%) | <0.01* |  | 3941 (26.76%) | 4030 (27.36%) | 0.24 |
| Anxiety disorders | 4659 (24.13%) | 4941 (25.59%) | <0.001* |  | 4138 (28.10%) | 4455 (30.25%) | <0.001* |
| BD | - | - | - |  | 4256 (28.90%) | 3847 (26.12%) | < 0.001* |
| Bipolar-related disorders (excluding BD) | 180 (0.93%) | 179 (0.93%) | 1.00 |  | 156 (1.06%) | 152 (1.03%) | 0.86 |
| MDD | 3742 (19.38%) | 4092 (21.19%) | <0.001* |  | - | - | - |
| Depressive disorders (excluding MDD) | 3665 (18.98%) | 3987 (20.65%) | <0.001* |  | 3566 (24.21%) | 3833 (26.03%) | <0.001* |
| Personality disorders | 1058 (5.48%) | 1013 (5.25%) | 0.08 |  | 848 (5.76%) | 728 (4.94%) | <0.01* |
| Schizophrenia | 4585 (23.75%) | 4095 (21.21%) | <0.001* |  | 3077 (20.89%) | 2568 (17.44%) | <0.001* |
| Schizophrenia spectrum disorders | 1613 (8.35%) | 1540 (7.98%) | 0.17 |  | 1318 (8.95%) | 1253 (8.51%) | 0.18 |
| Sleep-wake disorders | 1952 (10.11%) | 1947 (10.08%) | 0.95 |  | 1720 (11.68%) | 1782 (12.10%) | 0.26 |
| Trauma- and stressor-related disorders | 2407 (12.47%) | 2436 (12.62%) | 0.66 |  | 2108 (14.31%) | 2183 (14.82%) | 0.21 |
| Tardive dyskinesia | 15 (0.08%) | 23 (0.12%) | 0.26 |  | 13 (0.09%) | 16 (0.11%) | 0.71 |
| **Non-psychiatric comorbidities** |  |  |  |  |  |  |  |
| AIDS/HIV | 225 (1.17%) | 232 (1.20%) | 0.78 |  | 207 (1.41%) | 219 (1.49%) | 0.59 |
| Cancer | 337 (1.75%) | 357 (1.85%) | 0.47 |  | 351 (2.38%) | 377 (2.56%) | 0.35 |
| Cerebrovascular disease | 665 (3.44%) | 606 (3.14%) | 0.09 |  | 751 (5.10%) | 656 (4.45%) | <0.01 * |
| Congestive heart failure | 590 (3.06%) | 530 (2.74%) | 0.07 |  | 627 (4.26%) | 649 (4.41%) | 0.54 |
| Chronic pulmonary disease | 4295 (22.24%) | 4273 (22.13%) | 0.79 |  | 3618 (24.57%) | 3550 (24.10%) | 0.35 |
| Dementia | 285 (1.48%) | 234 (1.21%) | <0.05 * |  | 373 (2.53%) | 308 (2.09%) | <0.01* |
| Diabetes with chronic complication | 670 (3.47%) | 623 (3.23%) | 0.18 |  | 691 (4.69%) | 646 (4.39%) | 0.21 |
| Diabetes without chronic complication | 2616 (13.55%) | 2380 (12.33%) | <0.001* |  | 2267 (15.39%) | 2176 (14.77%) | 0.13 |
| Hemiplegia or paraplegia | 241 (1.25%) | 189 (0.98%) | <0.05* |  | 251 (1.70%) | 197 (1.34%) | <0.05* |
| Mild liver disease | 922 (4.78%) | 887 (4.59%) | 0.41 |  | 802 (5.45%) | 797 (5.41%) | 0.92 |
| Metastatic solid tumor | 49 (0.25%) | 63 (0.33%) | 0.22 |  | 57 (0.39%) | 71 (0.48%) | 0.24 |
| Myocardial infarction | 189 (0.98%) | 185 (0.96%) | 0.87 |  | 171 (1.16%) | 171 (1.16%) | 1.00 |
| Moderate or severe liver disease | 70 (0.36%) | 61 (0.32%) | 0.48 |  | 66 (0.45%) | 62 (0.42%) | 0.79 |
| Peptic ulcer disease | 133 (0.69%) | 148 (0.77%) | 0.40 |  | 131 (0.89%) | 122 (0.83%) | 0.61 |
| Peripheral vascular disease | 639 (3.31%) | 515 (2.67%) | <0.001* |  | 630 (4.28%) | 606 (4.11%) | 0.49 |
| Renal disease | 436 (2.26%) | 393 (2.04%) | 0.14 |  | 440 (2.99%) | 399 (2.71%) | 0.16 |
| Rheumatic disease | 282 (1.46%) | 271 (1.40%) | 0.66 |  | 295 (2.00%) | 306 (2.08%) | 0.68 |
| **Psychotherapy, n (%)** |  |  |  |  |  |  |  |
| Psychoanalysis | 1 (0.01%) | 2 (0.01%) | 1.00 |  | 0 (0.00%) | 0 (0.00%) | - |
| Psychotherapy in crisis | 38 (0.20%) | 39 (0.20%) | 1.00 |  | 32 (0.22%) | 31 (0.21%) | 1.00 |
| Psychotherapy non-crisis | 3123 (16.17%) | 3273 (16.95%) | <0.05* |  | 2697 (18.31%) | 2876 (19.53%) | <0.01* |
| **Psychiatric medication, n (%)** |  |  |  |  |  |  |  |
| Antidepressant | 10957 (56.75%) | 11151 (57.75%) | <0.01* |  | 9893 (67.17%) | 9991 (67.84%) | 0.06 |
| Anticholinergic | 2093 (10.84%) | 1656 (8.58%) | <0.001* |  | 1399 (9.50%) | 1011 (6.86%) | <0.001* |
| Sedative | 2848 (14.75%) | 2799 (14.50%) | 0.47 |  | 2427 (16.48%) | 2388 (16.21%) | 0.52 |
| Mood stabilizer | 8343 (43.21%) | 7669 (39.72%) | <0.001* |  | 5420 (36.80%) | 5133 (34.85%) | <0.001* |
| Anxiety medication | 6587 (34.12%) | 6846 (35.46%) | <0.01* |  | 5549 (37.68%) | 5723 (38.86%) | <0.05 * |
| ADHD medication | 1642 (8.50%) | 1611 (8.34%) | 0.55 |  | 887 (6.02%) | 907 (6.16%) | 0.62 |

ADHD: attention deficit hyperactivity disorder; AIDS/HIV: acquired immune deficiency syndrome/human immunodeficiency virus infection; BD: bipolar disorder; CCI: Charlson comorbidity index; FFS: fee-for-service; HMO: health maintenance organization; MDD: major depressive disorder; SD: standard deviation. **P*<0.05
