# Supplementary material for: Hospital utilization rates following antipsychotic dose reduction in mood disorders: implications for treatment of tardive dyskinesia
Source: BMC Psychiatry. 2020 Jul 11;20:365. doi: 10.1186/s12888-020-02748-0 (PMC7353680; doi:10.1186/s12888-020-02748-0)
Supplement: Supplementary file 3 — Additional file 3. Dose Distributions Among Patients in the BD and MDD Groups During the ≤90-day Stable Dose Period Prior to the Index Date for the 10 Most Commonly Used Antipsychotic Medications. [file 12888_2020_2748_MOESM3_ESM.docx]

**Additional File 3.** **Dose Distributions Among Patients in the BD and MDD Groups During the ≤90-day Stable Dose Period Prior to the Index Date for the 10 Most Commonly Used Antipsychotic Medications.**

|  | **BD** | | | | **MDD** | | | |
| --- | --- | --- | --- | --- | --- | --- | --- | --- |
|  | **Cases** | | **Controls** | | **Cases** | | **Controls** | |
| **Drug** | **N** | **Dose (mg/day),**  **mean ± SD** | **N** | **Dose (mg/day), mean ± SD** | **N** | **Dose (mg/day),**  **mean ± SD** | **N** | **Dose (mg/day),**  **mean ± SD** |
| Quetiapine | 8624 | 404 ± 259 | 6580 | 266 ± 227 | 6713 | 343 ± 247 | 5231 | 218 ± 197 |
| Aripiprazole | 3710 | 22 ± 31 | 5485 | 14 ± 25 | 3278 | 18 ± 31 | 4850 | 11 ± 23 |
| Risperidone | 3745 | 5 ± 6 | 3616 | 4 ± 4 | 2700 | 4 ± 5 | 2620 | 3 ± 3 |
| Olanzapine | 2180 | 19 ± 11 | 2231 | 14 ± 10 | 1361 | 17 ± 11 | 1295 | 12 ± 9 |
| Ziprasidone | 1859 | 135 ± 56 | 1409 | 108 ± 56 | 1093 | 130 ± 57 | 705 | 103 ± 57 |
| Lurasidone | 752 | 85 ± 36 | 1413 | 60 ± 33 | 559 | 83 ± 36 | 931 | 58 ± 33 |
| Paliperidone | 840 | 62 ± 63 | 1039 | 50 ± 66 | 524 | 58 ± 66 | 605 | 52 ± 65 |
| Haloperidol | 704 | 23 ± 22 | 641 | 17 ± 17 | 468 | 21 ± 32 | 426 | 14 ± 17 |
| Clozapine | 488 | 368 ± 177 | 247 | 368 ± 190 | 270 | 364 ± 174 | 124 | 342 ± 187 |
| Asenapine | 273 | 17 ± 7 | 354 | 13 ± 6 | 156 | 17 ± 9 | 202 | 13 ± 6 |

BD: bipolar disorder; MDD: major depressive disorder; mg: milligrams; SD: standard deviation
