# Supplementary material for: Hospital utilization rates following antipsychotic dose reduction in mood disorders: implications for treatment of tardive dyskinesia
Source: BMC Psychiatry. 2020 Jul 11;20:365. doi: 10.1186/s12888-020-02748-0 (PMC7353680; doi:10.1186/s12888-020-02748-0)
Supplement: Supplementary file 4 — Additional file 4. Dose Reduction Percentiles for the 10 Most Commonly Used Antipsychotic Medications Among Patients in the BD and MDD Groups. [file 12888_2020_2748_MOESM4_ESM.docx]

**Additional File 4. Dose Reduction Percentiles for the 10 Most Commonly Used Antipsychotic Medications Among Patients in the BD and MDD Groups.**

|  |  |  |  |  |  |  |  |  | |  |  |  |  |  |  |
| --- | --- | --- | --- | --- | --- | --- | --- | --- | --- | --- | --- | --- | --- | --- | --- |
|  | BD | | | | | | | | MDD | | | | | | |
|  |  |  |  |  |  |  |  |  |  |  |  |  |  |  |  |
| **Drug** | **N** | **Mean** | **10%** | **25%** | **50%** | **75%** | **90%** | **N** | | **Mean** | **10%** | **25%** | **50%** | **75%** | **90%** |
| Quetiapine | 8624 | 48.40% | 25.00% | 33.30% | 50.00% | 66.70% | 75.00% | 6713 | | 48.80% | 25.00% | 33.30% | 50.00% | 65.30% | 75.00% |
| Aripiprazole | 3710 | 47.60% | 25.00% | 33.30% | 50.00% | 60.00% | 75.00% | 3278 | | 48.70% | 25.00% | 33.30% | 50.00% | 60.00% | 71.40% |
| Risperidone | 3745 | 48.00% | 25.00% | 33.30% | 50.00% | 60.00% | 75.00% | 2700 | | 48.00% | 25.00% | 33.30% | 50.00% | 50.00% | 75.00% |
| Olanzapine | 2180 | 46.70% | 25.00% | 33.30% | 50.00% | 60.00% | 75.00% | 1361 | | 46.90% | 25.00% | 33.30% | 50.00% | 60.00% | 71.80% |
| Ziprasidone | 1859 | 45.70% | 25.00% | 33.30% | 50.00% | 50.00% | 75.00% | 1093 | | 45.10% | 25.00% | 33.30% | 50.00% | 50.00% | 75.00% |
| Lurasidone | 752 | 47.20% | 25.00% | 33.30% | 50.00% | 50.00% | 66.70% | 559 | | 46.40% | 25.00% | 33.30% | 50.00% | 50.00% | 66.70% |
| Paliperidone | 840 | 56.60% | 25.00% | 33.30% | 50.00% | 90.00% | 95.00% | 524 | | 56.00% | 25.00% | 33.30% | 50.00% | 85.00% | 95.00% |
| Haloperidol | 704 | 52.80% | 25.00% | 33.30% | 50.00% | 70.40% | 83.30% | 468 | | 51.20% | 25.00% | 33.30% | 50.00% | 66.70% | 80.00% |
| Clozapine | 488 | 47.20% | 16.70% | 23.10% | 40.00% | 75.00% | 87.50% | 270 | | 48.40% | 18.00% | 25.00% | 41.60% | 75.00% | 87.50% |
| Asenapine | 273 | 49.80% | 33.30% | 50.00% | 50.00% | 50.00% | 66.70% | 156 | | 49.70% | 33.30% | 50.00% | 50.00% | 50.00% | 66.70% |
|  |  |  |  |  |  |  |  |  | |  |  |  |  |  |  |

BD: bipolar disorder; MDD: major depressive disorder.
