# Supplementary figures and images for: Hospital utilization rates following antipsychotic dose reduction in mood disorders: implications for treatment of tardive dyskinesia
Source: BMC Psychiatry. 2020 Jul 11;20:365. doi: 10.1186/s12888-020-02748-0 (PMC7353680; doi:10.1186/s12888-020-02748-0)

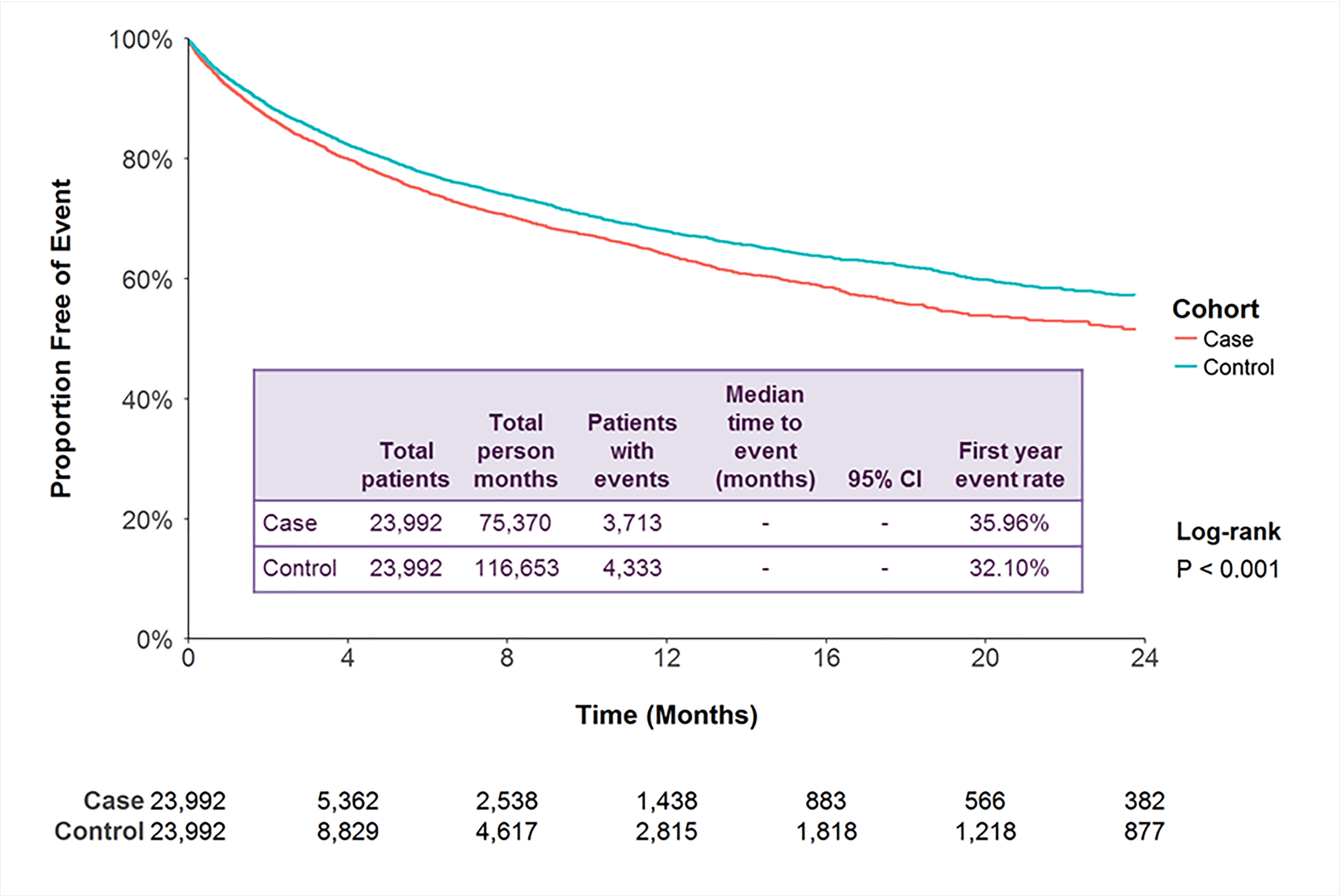

Supplement: Supplementary file 5 — Additional file 5. Psychiatric Admission With ≥10% Antipsychotic Dose Reduction in the BD Group. Patient claims were analyzed for psychiatric admissions related to BD for ≥10% dose reductions of antipsychotic medication. Outcomes for case and control cohorts were assessed using Kaplan–Meier analysis and compared using a log-rank test. The number of patients at risk is represented for each time point. Case and control cohorts for ≥10%, N = 23,992 each. BD: bipolar disorder; CI: confidence interval. [file 12888_2020_2748_MOESM5_ESM.tif]

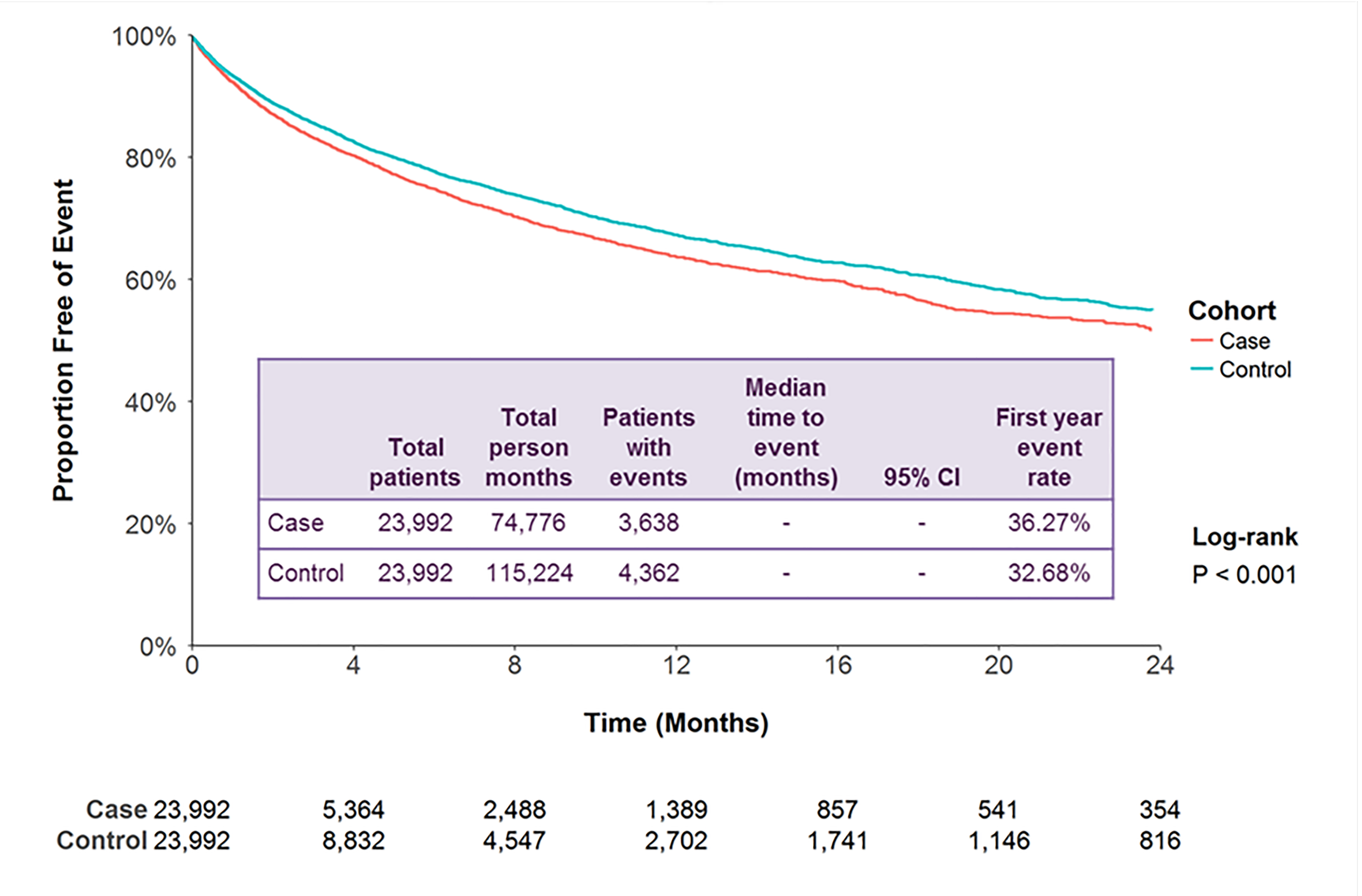

Supplement: Supplementary file 6 — Additional file 6. All-Cause Inpatient Admissions Among Patients With ≥10% Antipsychotic Dose Reduction in the BD Group. Patient claims were analyzed for all-cause inpatient admissions related to BD for ≥10% dose reductions of antipsychotic medication. Outcomes for case and control cohorts were assessed using Kaplan–Meier analysis and compared using a log-rank test. The number of patients at risk is represented for each time point. Case and control cohorts for ≥10%, N = 23,992 each. BD: bipolar disorder; CI: confidence interval; IP: inpatient. [file 12888_2020_2748_MOESM6_ESM.tif]

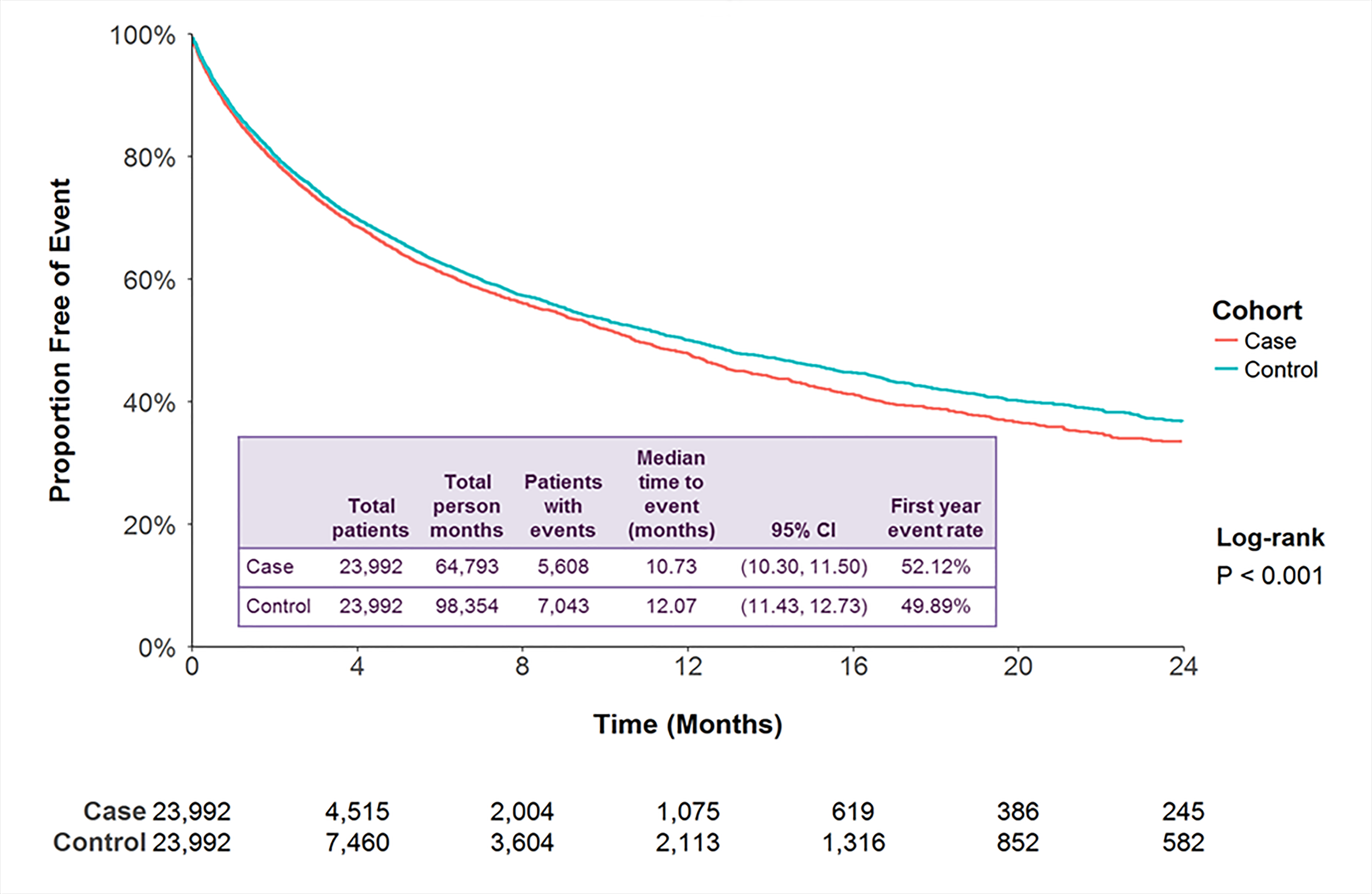

Supplement: Supplementary file 7 — Additional file 7. All-Cause Emergency Room Visits Among Patients With ≥10% Antipsychotic Dose Reduction in the BD Group. Patient claims were analyzed for all-cause emergency room admissions related to BD for ≥10% dose reductions of antipsychotic medication. Outcomes for case and control cohorts were assessed using Kaplan–Meier analysis and compared using a log-rank test. The number of patients at risk is represented for each time point. Case and control cohorts for ≥10%, N = 23,992 each. BD: bipolar disorder; CI: confidence interval; ER: emergency room. [file 12888_2020_2748_MOESM7_ESM.tif]

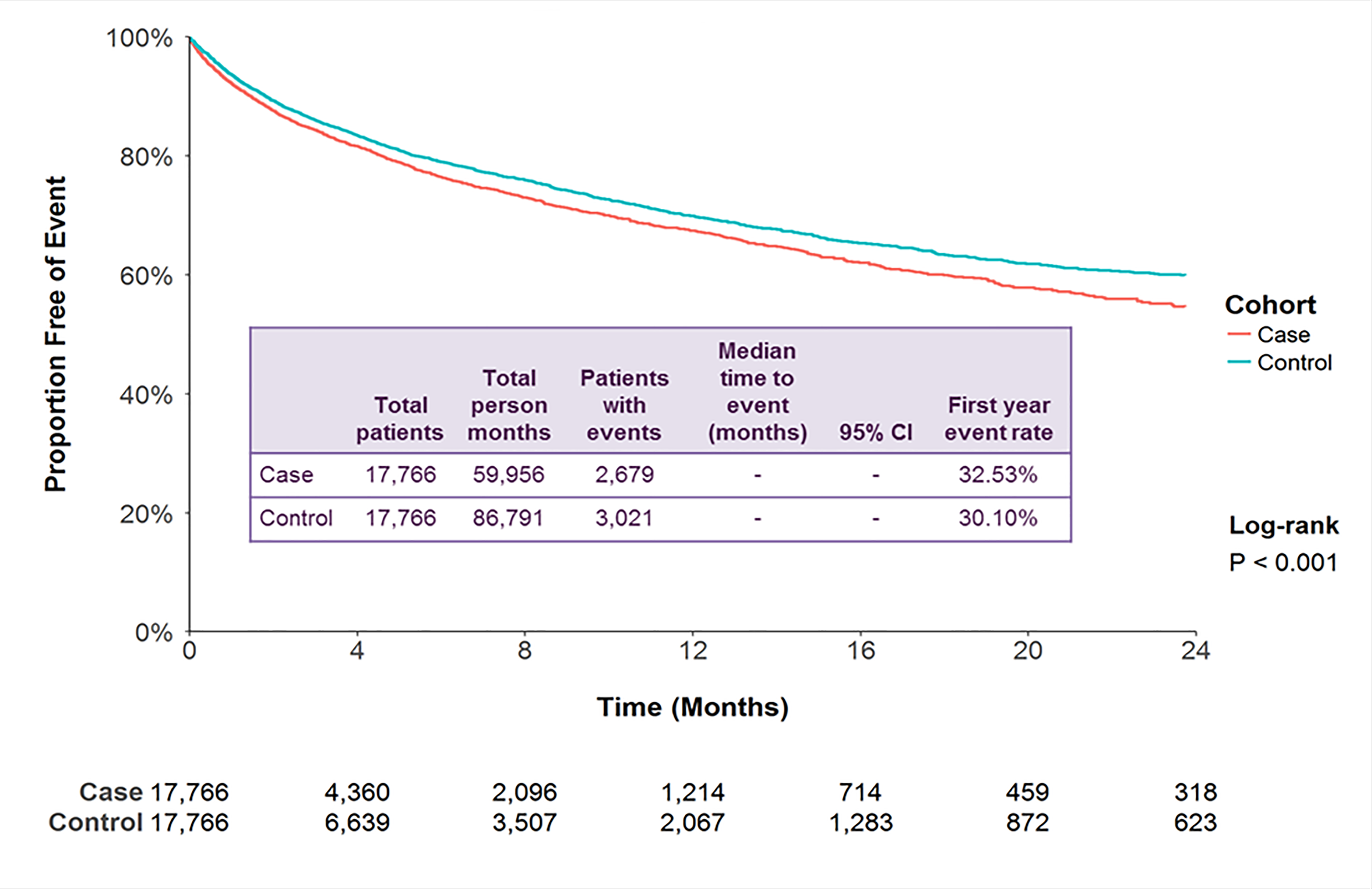

Supplement: Supplementary file 8 — Additional file 8. Psychiatric Admission Among Patients With ≥10% Antipsychotic Dose Reduction in the MDD Group. Patient claims were analyzed for psychiatric admissions related to MDD for ≥10% dose reductions of antipsychotic medication. Outcomes for case and control cohorts were assessed using Kaplan–Meier analysis and compared using a log-rank test. The number of patients at risk is represented for each time point. Case and control cohorts for ≥10%, N = 17,766 each. CI: confidence interval; MDD: major depressive disorder. [file 12888_2020_2748_MOESM8_ESM.tif]

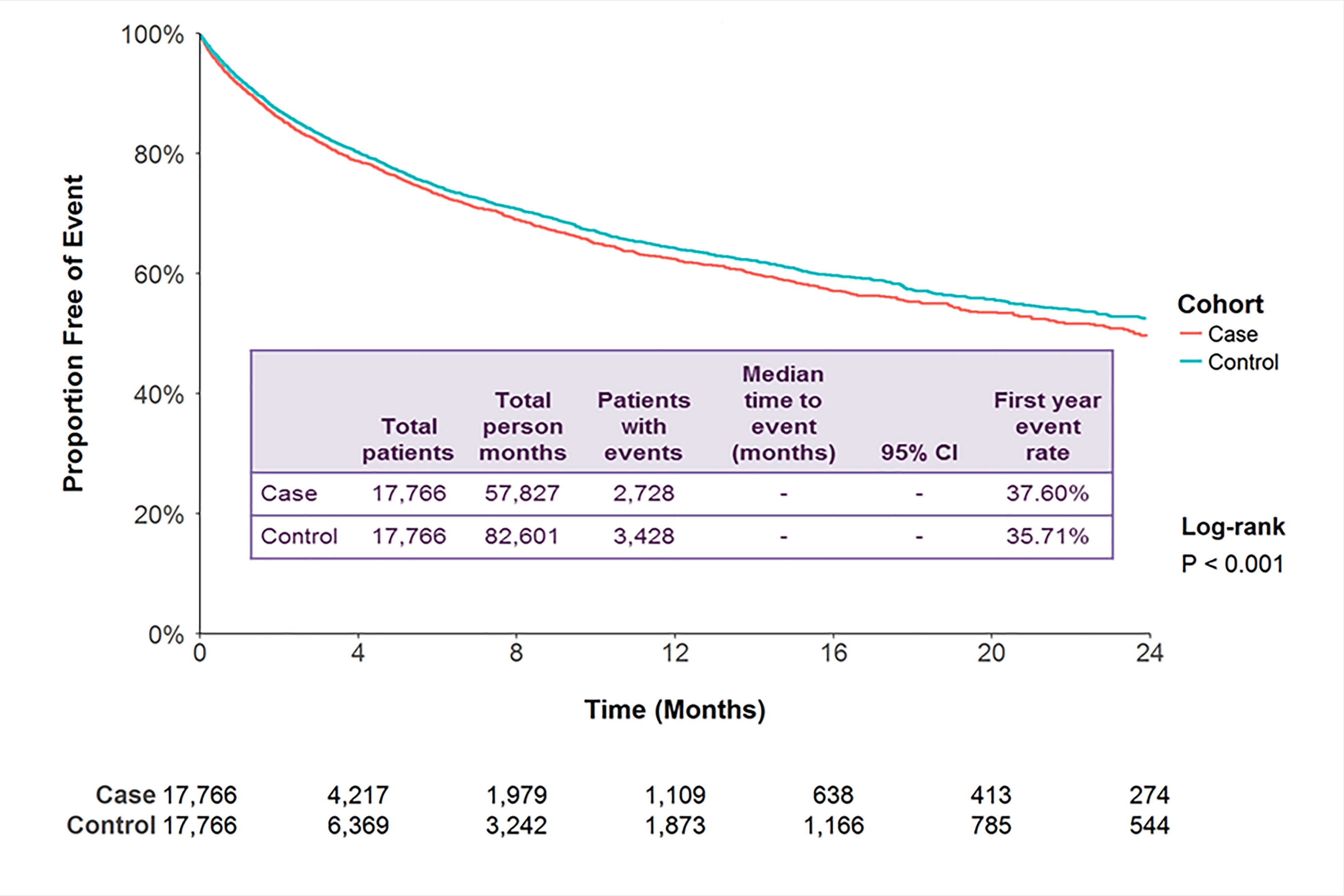

Supplement: Supplementary file 9 — Additional file 9. All-Cause inpatient Admissions Among Patients With ≥10% Antipsychotic Dose Reduction in the MDD Group. Patient claims were analyzed for all-cause inpatient admissions related to MDD for ≥10% dose reductions of antipsychotic medication. Outcomes for case and control cohorts were assessed using Kaplan–Meier analysis and compared using a log-rank test. The number of patients at risk is represented for each time point. Case and control cohorts for ≥10%, N = 17,766 each. CI: confidence interval; IP: inpatient; MDD: major depressive disorder. [file 12888_2020_2748_MOESM9_ESM.tif]

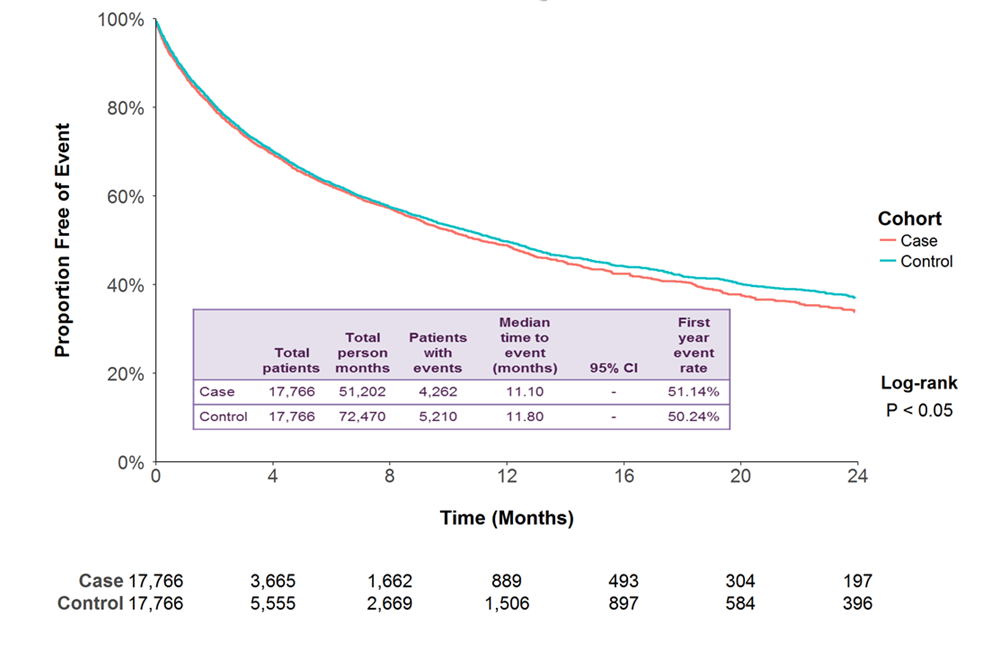

Supplement: Supplementary file 10 — Additional file 10. All-Cause ER Visits Among Patients With ≥10% Antipsychotic Dose Reduction in the MDD Group. Patient claims were analyzed for all-cause emergency room admissions related to MDD for ≥10% dose reductions of antipsychotic medication. Outcomes for case and control cohorts were assessed using Kaplan–Meier analysis and compared using a log-rank test. The number of patients at risk is represented for each time point. Case and control cohorts for ≥10%, N = 17,766 each. CI: confidence interval; ER: emergency room; MDD: major depressive disorder. [file 12888_2020_2748_MOESM10_ESM.tif]
